# Supplementary material for: Rehospitalization for pneumonia after first pneumonia admission: Incidence and predictors in a population-based cohort study
Source: PLoS One. 2020 Jun 30;15(6):e0235468. doi: 10.1371/journal.pone.0235468 (PMC7326167; doi:10.1371/journal.pone.0235468)
Supplement: S1 Table — Lombardy region, Italy, 2003–2012. (DOC) [file pone.0235468.s001.doc]

**Appendix**

**Supplemental table S1**. Diagnostic and medication codes considered in the current study

| **DISEASES** | **ICD-9 CM code** |
| --- | --- |
| **Outcome** |  |
| Pneumonia |  |
| Viral pneumonia | 480.x |
| Pneumococcal Pneumonia | 481.x |
| Other bacterial pneumonia | 482.x |
| Pneumonia due to other specific microorganisms | 483.x |
| Pneumonia otherwise classified | 484.x |
| Bronchopneumonia without specified organism | 485.x |
| Pneumonia, not specified organism | 486.x |
| Flu with pneumonia | 487.0, 488.x |
|  |  |
| **Covariates/Exclusion criteria** |  |
| Immunodeficiency diseases |  |
| Leukemia | 204.0 – 208.9 |
| White blood cells disease | 288.x |
| AIDS | 042 |
| Splenectomy | 41.5 |
| Chest x-ray | 87.44 |
| Chest CT scan | 87.41 |
| Blood culture | 90.5, 790.7, 795.39 |
| Chronic lung diseases | 518.89, 496.x |
| COPD | 491.x, 492.x, 496.x |
| Bronchiectasis | 494 |
| Asthma | 493.x |
| Diffuse interstitial lung disease | 508.1, 515, 516.3, 518.89, 714.81, 770.7 |
| Cystic fibrosis | 277.0 |
| OSAS | 327.23 |
| Pulmonary hypertension | 416.0 |
| Cancer | 140.x-208.x |
| Cardiovascular disease | 390.x – 459.x |
| Diabetes | 250.x |
| Chronic kidney failure | 585.x |
| Cirrhosis | 517.5 |
| Parkinson’s disease | 322.0x |
| Epilepsy | 345.x |
| Dementia | 290.x, 294.x, 331.x, 334.x, 335.x |
| Depression | 296.20, 296.21, 296.22, 296.23, 296.25, 296.26, 296.30, 296.31, 296.32, 296.33, 296.35, 296.36, 300.4, 309.0, 309.1, 311 |
|  |  |
| **DRUGS** | **ATC code** |
|  |  |
| **Covariates/Exclusion criteria** |  |
| Antibiotic therapy | J01, J04X |
| Antifungal therapy | J02 |
| Antiviral therapy | J05 |
| Immunosuppressive therapy | L01, L04 |
| Calcineurin inhibitors | L04AD |
| Sirolimus | L04AA10 |
| Methotrexate | L04AX03 |
| Systemic Corticosteroid therapy | H02 |
| Blood glucose lowering agents | A10B |
| Antiarrhythmics | C01B |
| Digoxin | C01AA05 |
| Benzodiazepines | N03AE, N05BA, N05CD, N05CF |
| Antiepileptics | N03 |
| Antihypertensives | C02, C03, C07, C08, C09 |
| Neuroleptics | N05A |
| Antidepressants | N06A |
| NSAIDs | M01A |
| Antineoplastics | L01 |
| Asthma and COPD drugs | R03 (without R03BA) |
| Inhaled corticosteroids | R03BA (glucocorticoids) |

AIDS =acquired immune deficiency syndrome

COPD = Chronic obstructive pulmonary disease

OSAS = Obstructive sleep apnoea syndrome

NSAIDs = non steroidal anti- inflammatory drugs
