# Supplementary material for: Rehospitalization for pneumonia after first pneumonia admission: Incidence and predictors in a population-based cohort study
Source: PLoS One. 2020 Jun 30;15(6):e0235468. doi: 10.1371/journal.pone.0235468 (PMC7326167; doi:10.1371/journal.pone.0235468)
Supplement: Attachment — Submitted filename: Rebuttal Letter.doc [file pone.0235468.s002.doc]

Thank you very much indeed for giving us the opportunity to submit a revised version of our manuscript. We have addressed all the points very reasonably raised by the Reviewers and amended our manuscript accordingly. We believe this has increased the quality of our article.

Please find below a point-by-point reply to the Reviewers comments, as requested.

We hope our revised manuscript will be considered for publication in CHEST in its present form.

Sincerely yours

Paola Faverio (for the Authors)

**Reviewer 1**

*This study shows the factors associated with readmissions in a large cohort of patients with pneumonia. Also, this study adds evidence about complexity medication regimens as a possible predictive factor for readmission.*

*The follow-up of patients included early and late readmission (30 and 90 days). Unfortunately, the analysis of patients who had a late readmission were not presented. According to your study aimed, information should be provided regarding patient characteristics and associated factors with late readmissions.*

**Reply:** We thank the reviewer for this suggestion. As the reviewer said, in our study the follow-up of patients included as outcome the first hospitalization for pneumonia; these rehospitalizations included both early (within 30 days of discharge after the first pneumonia admission) and late (between 31 and 90 days after discharge) readmissions for pneumonia. Therefore, the aim of our study was to evaluate the incidence and predictors of early and late readmissions due to pneumonia.

However, from a first explorative analysis, as reported at page 9, lines 5-8 of the old version of our manuscript, it resulted that, from 31 to 90 days after the index date, only 47 patients (0.02%) experienced a late readmission for pneumonia. Thus, considering that the rate of late readmissions is very low, statistical analyses were carried out only for early readmissions. Statistical analyses were not carried out also for late readmissions for pneumonia because a lack of statistical power would have been observed, mainly due to the low number of events considered, which would not have allowed to highlight appreciable statistically significant differences in the predictor variables.

Then, considering the potential lack of statistical power that could have occurred analyzing late pneumonia readmissions, and also taking into consideration the various suggestions raised by the reviewer #2 who proposed not to include late readmissions in the main analyses, we decided to remove from the main analyses the readmissions that occurred beyond 30 days after the index date. We revised the paper focusing on the early readmissions (i.e., those within 30 days after the index discharge for pneumonia), being these early readmissions the standard that most other research in this area examined.

However, we mentioned late readmissions a few times in the manuscript to broaden the discussion and introduce potential future developments of this research.

*The study data were retrieved from the health care utilization database of NHS. Community-acquired pneumonia and hospital-acquired pneumonia were included? Please, add the pneumonia criteria and ICD-9CM codes included of index admission. Interestingly, perhaps you have included information about type of pneumonia and causative microorganism.*

**Reply:** As the Reviewer suggested, our study is based on HealthCare Utilization (HCU) databases on Lombardy region, and all the 266,766 residents in Lombardy region assisted by the NHS wo had experienced at least one hospital admission with primary or secondary diagnosis of pneumonia, during the years 2003-2012, were firstly identified to be included in the study. Hospitalizations with pneumonia as secondary diagnosis were also considered because both the primary and secondary diagnoses contributed decisively in the hospital admission decision. As diagnosis of pneumonia for the index hospitalization, we considered those that had been recorded with an ICD-9-CM code from 480.x to 488.x, excluding 487.x but including 4870.x.

We considered also hospital admissions with primary or secondary diagnosis with the ICD-9-CM code 486.x, so we could reasonably state that both Hospital-Acquired and Community-Acquired Pneumonia (HAP and CAP, respectively) has been included in the index hospitalization, since the ICD-9-CM code 486.x is used to record also community acquired pneumonia, healthcare associated pneumonia and hospital acquired pneumonia.

Furthermore, since the major outcome was the first rehospitalization for pneumonia within 30 days after the discharge from the first pneumonia admission, both HAP and CAP had been included also in this category.

As requested by the reviewer, we added in the manuscript the pneumonia ICD-9-CM codes that were considered for the identification of the index hospitalization [Materials and Methods section, Setting subsection, page 6, lines 19-20; Outcome subsection, page 7, lines 9-10].

Further information about type of pneumonia and causative microorganism were not available, since one of the major limitations of our study is that the information recorded in HCU databases did not include clinical data as causative microorganism, etc.

*You need explain if a collinearity analysis was performed. Why did you not show the use of statins (Table 1)?*

**Reply:** We have considered collinearity in the context of a statistical model that is used to estimate the relationship between one response variable and a set of predictor variables; multicollinearity occurs when there are high correlations among predictor variables, leading to unreliable and unstable estimates of regression coefficients. We performed a collinearity analysis considering most widely-used diagnostic for multicollinearity, the Variance Inflation Factor (VIF). VIF has been calculated for each covariate by performing a linear regression model of that predictor on all the other covariates. The VIF has been calculated using the following formula: 1/(1-R^2^); with R^2^ estimated from the above-mentioned linear regression model. This parameter, i.e., VIF, estimates how much the variability of a coefficient is inflated because of linear dependence with other predictor variables. The VIF has a lower bound of 1 but no upper bound; in order to classify VIF as “High”, we fixed the threshold of 3. We included in the main analysis only those predictor variables that did not have high VIFs.

According to the reviewer’s suggestion, information regarding the previous use of lipid-lowering and antihypertensive medications has been added in Table 1 [pages 23-24]

*Tables, figures, and references need a review (references duplicate nº 5 and 14)*

**Reply:** According to the suggestion of the reviewer, we had carefully read the manuscript many times, particularly focusing on Tables, Figures and References. References duplicated has been corrected, and several modifications has been apported to tables, figures, figures legends and references’ format. Furthermore, Table 1 has been consistently modified [pages 23-24]. Also, Table 2 [pages 25] and Figure 2 were consistently revised.

*The introduction and discussion need a little information regarding frail elderly patients.*

**Reply:** We thank the reviewer for this advice. According to his suggestion, the topic of frail patients has been introduced and discussed thoughtfully in the manuscript, and it has been now reported in the Introduction and Discussion section [Introduction, Page 5, Lines 1-8; Discussion, Page 14, Lines 4-11; Page 16, lines 23-24; Page 17, lines 1-2]

**Reviewer 2**

The authors present a retrospective review of a large cohort of patients admitted with pneumonia to multiple hospitals in Northern Italy between 2003 and 2012 in which they assess the rate of pneumonia-specific readmission and its contributing cofactors. As opposed to most other studies on pneumonia readmission rates, the authors focus on related, pneumonia-specific readmissions, rather than all-cause readmissions. This is a strength of the study. Limitations include the significant delay between data collection and manuscript.

**Major critiques**

*The authors should explain why they feel it is relevant to consider readmissions that occur beyond 30 days in their analysis. Intuitively it seems that these so called “late readmissions” are unrelated to factors involved in the original case. Furthermore, the rate of late readmissions is very low. Unless the authors can add further justification for including this in the paper, I would suggest* *removal and focusing on the readmissions within 30 days which is the standard most other research in this area examines.*

**Reply:** We thank the reviewer for this suggestion. As the reviewer said, the aim of our study was to evaluate the incidence and predictors of early and late readmissions due to pneumonia.

However, from a first explorative analysis, as reported at page 9, lines 5-8 of the old version of our manuscript, it resulted that, from 31 to 90 days after the index date, only 47 patients (0.02%) experienced a late readmission for pneumonia. Thus, considering that the rate of late readmissions is very low and the potential lack of statistical power that could have occurred analyzing separately late pneumonia readmissions, according to the reviewer suggestion we decided to remove from the main analyses the readmissions that occurred beyond 30 days after the index date. We revised the paper focusing on the early readmissions (i.e., those within 30 days after the index discharge for pneumonia), being these early readmissions the standard that most other research in this area examined.

However, we mentioned late readmissions a few times in the manuscript to broaden the discussion and introduce potential future developments of this research.

*The data being analyzed in this study is currently 8 – 17 years old. Certainly, temporal changes in the care of hospitalized patients have transpired, and* *the fact that the conclusions are based on old data should be clarified as a limitation in the discussion sections.* *Ideally the authors would frame their results against comparative readmission rates from more recent data and put their results into context. Unfortunately, few studies exist with pneumonia specific readmission rates, but if the authors have access to all-cause* *30-day readmission rates, they could make the case for whether temporal trends suggest increasing, decreasing or stable rates compared to the study period.*

**Reply:** we thank the reviewer for this advice. We discussed this topic in the Discussion section regarding the limitations of the study [page 16, lines 16-21].

Furthermore, as suggested by the reviewer, all-cause 30-day readmission rate was calculated and widely discussed along the manuscript [page 10, lines 4-5; page 13, lines 5-10; page 16, lines 16-21].

As suggested by the reviewer the data analyzed in this study relate to several years ago and are not updated, for this reason our study may not have been able to include some changes that have recently occurred in the care of hospitalized patient for pneumonia. However, our all-case 30-day readmission rates after a first pneumonia hospitalization agree with those reported in recent observational studies, ranging from 8.6 to 25% [6,8,19-20], thus we can likely suppose that all-case 30-day readmission rates after a first pneumonia hospitalization remained stable

*In Table 1, there appears to be significant differences in various comorbidities between readmitted and not readmitted patients; this is pointed out in line 15 on p 9. However, when adjusted hazard ratios are calculated (Table 2) only renal disease appears to be a predictor. In the Results section (p10, line 4-7) the authors note that use of meds for chronic lung disease were associated with increased readmission risk, but chronic lung disease as a comorbidity was not significant.* *Aren't some of these meds in fact "proxies" for the underlying chronic medical conditions? The authors should discuss these dichotomies and the implications for the limitations of their data in the discussion section.*

**Reply:** We thank the reviewer for this suggestion. Reading the manuscript and checking the results, we found that a result was incorrectly reported: in Table 2 the Hazard ratios (HR), and relative 95% confidence intervals (CI), of early rehospitalizations for pneumonia for patients having Chronic lung disease was not 0.95 (0.88-1.02), but the correct estimates (now reported in Table 2) are 1.12 (1.03-1.25). The appropriate and related changes were therefore made in the text [Page 7, lines 24-25; Page 11, lines 5-6; Table 2; Figure 2].

*The paper would benefit from more careful proofreading and correction of grammatical errors, and some effort to improve the clarity of writing.*

**Reply:** We thank the reviewer for this advice. According to this suggestion, we had the proofs of the manuscript corrected by a professional translator Ms. Mary McKenney.

*Data Table 1 lacks column headings, these should be added.*

**Reply:** According to the reviewer’s suggestion, Table 1 has been consistently modified, and also column headings have been added.

**Minor critiques**

*Late readmissions are mentioned in the introduction but given the lack of clear clinical relevance and the exceedingly small number identified by the author, I think the paper would be more effective if this detail was omitted throughout the manuscript.*

**Reply:** We thank the reviewer for this suggestion. According to this, as better explained in the response to his first suggestion in the **Major critiques** section, we revised the paper focusing on the early readmissions (i.e., those within 30 days after the index discharge for pneumonia), being these the standard that most other respiratory medicine research examines. However, we mentioned late readmissions a few times in the manuscript to broaden the discussion and introduce potential future developments of this research.

*The statement about readmission penalties in the US is inaccurate in that it refers to the “private health insurance system.” In fact, 30-day readmission penalties in the US are imposed by Medicare which is a federally funded health insurance program for adults age 65 and older. It is not private insurance.*

**Reply:** We agree with the suggestion of the reviewer and the statement about readmission penalties in the US previously referring to the “private health insurance system” has been rewritten [page 5, lines 22-23; page 6, lines 1-2].

*Why does p 7 line 16 state that info was collected about prior admissions for pneumonia when this group of patients was excluded according to the text and Figure 1?*

**Reply:** We thank the reviewer for his suggestion, this step of the cohort selection procedure is not very clear and misleading. According to the reviewer suggestion the sentence “*previous admissions for pneumonia (up to 6 months before the index date)*” was deleted.

We still want to highlight that patients who experienced a hospitalization for pneumonia in the 6 months before the index date has been excluded; with this cautionary criterium we ensured the inclusion in the cohort of patients for whom the index admission was not a rehospitalization after prior recent pneumonia hospital admission, avoiding a potential misclassification of the outcome.

*I had difficulty linking a clinical relationship to pneumonia readmission rates and some of the factors on the list of clinical characteristics detailed on p7 line 20. Can the authors explain why they chose to include thrombocytosis and pressure ulcer as clinical covariates?*

**Reply:** We thank the reviewer for this comment. We made a selection of covariates starting from the scientific literature (*Weinreich M et al.* Predicting the Risk of Readmission in Pneumonia: A Systematic Review of Model Performance. Ann Am Thorac Soc. 2016 Sep;13(9):1607-14; *Makam AN et al.* Predicting 30-Day Pneumonia Readmissions Using Electronic Health Record Data Journal of Hospital Medicine 2017 Vol 12. No 4). Furthermore, thrombocytosis is often used as a proxy of activated inflammatory state and pressure ulcers are more common in frail patients, a population analyzed in our study.

*P9 line 5-8: More reason to exclude mention of the concept of late readmissions in this analysis. It adds little other than distracting from the main findings.*

**Reply:** According to the reviewer this suggestion, and the previous ones, this sentence has been deleted.

*Figure 1 – graphic is blurred.*

**Reply:** According to the reviewer’s suggestion, Figure 1 has been modified and saved in a new format, in order to be clearer and sharper.

For a better view of the Figure 1, from the PDF file submitted to the Editorial Manager of the Journal, we please the reviewer not to view the Figure 1 directly from the PDF of the submission, but by going to the link (top right, “*Click here to access / download ; Figure; Figure 1. Flow-chart.tiff*”) and view the image after having downloaded it from the corresponding webpage; by doing this, the resolution of the image of Figure 1 will be higher.

*Page 12 line 22, 23 – further explanation as to why inhaled bronchodilators were associate with increased risk of admission whereas ICS-Bronchodilator combination was not.*

**Reply:** We thank the reviewer for this comment. This result may be due to the effect of the association with inhaled steroids. In fact, inhaled steroids were not associated with an increased risk of hospital readmission.

However, this is a population study based on HCU databases and not a pharmacological study, therefore the effect of individual molecules or associations cannot be assessed.

*Figure 2 – No point estimate labeled for younger or older patients, therefore unable to visually compare point estimates between both groups*

**Reply:** According to the reviewer suggestion, Figure 2 has been modified labelling the point estimates for younger and older patients.

*P13 line 1-6, the authors follow discussion of a finding that states that inhaled steroids were not a predictor of readmission with a reference to a study that found that inhaled steroids are associated with higher risk of developing pneumonia. Was this a study about pneumonia readmission rates or primary episodes of pneumonia? If the latter, then the link between the 2 thoughts does not logically connect. Consider rewriting or removing.*

**Reply:** We thank the reviewer for this observation and removed from the text the citation.

*Since you have a separate paragraph addressing the relationship of systemic steroids and pneumonia readmissions (p13 line 17-18), consider removing mention of steroids in line 7 and just focus on ASA in this paragraph.*

**Reply:** We agree with the reviewer on this suggestion and changed the text accordingly.

**Response to Journal Requirements:**

1. Please ensure that your manuscript meets PLOS ONE's style requirements, including those for file naming. The PLOS ONE style templates can be found at <http://www.plosone.org/attachments/PLOSOne_formatting_sample_main_body.pdf> and <http://www.plosone.org/attachments/PLOSOne_formatting_sample_title_authors_affiliations.pdf>

**Response:** we revised the style and file names.

2. We noticed you have some minor occurrence(s) of overlapping text with the following previous publication(s), which needs to be addressed:

<https://doi.org/10.1016/j.ejim.2017.09.023> (Good adherence...) <https://doi.org/10.1002/pds.4206> (exposure to statins...)

<https://doi.org/10.1186/s12883-017-0796-3> (TIA stroke..)

In your revision ensure you cite all your sources (including your own works), and quote or rephrase any duplicated text outside the Methods section. Further consideration is dependent on these concerns being addressed.

**Response:** According to the Journal Requirements several sentences in the manuscript had been fully rewritten. Furthermore, new references have been added in the Material and Methods, Setting subsection.

3. In the ethics statement in the manuscript and in the online submission form, please provide additional information about the patient records/samples used in your retrospective study. Specifically, please ensure that you have discussed whether all data/samples were fully anonymized before you accessed them and/or whether the IRB or ethics committee waived the requirement for informed consent. If patients provided informed written consent to have data/samples from their medical records used in research, please include this information.

**Response:** According to the Journal Requirements we have fully rewritten the Ethical approval and consent to participate section and moved this section in the Methods section of our manuscript.

4. Thank you for stating the following in the Competing Interests section:
   "I have read the journal's policy and the authors of this manuscript have the following competing interests: G.C. received research support from the European Community (EC), the Italian Medicines Agency (AIFA), and the Italian Ministry of Education, Universities and Research (MIUR). He took part in a variety of projects that were funded by pharmaceutical companies (Novartis, GSK, Roche, AMGEN and BMS). He also received honoraria as a member of the Advisory Board of Roche. No other potential conflicts of interest were declared."

Please confirm that this does not alter your adherence to all PLOS ONE policies on sharing data and materials, by including the following statement: "This does not alter our adherence to PLOS ONE policies on sharing data and materials.” (as detailed online in our guide for authors <http://journals.plos.org/plosone/s/competing-interests>). If there are restrictions on sharing of data and/or materials, please state these. Please note that we cannot proceed with consideration of your article until this information has been declared.

Please include your updated Competing Interests statement in your cover letter; we will change the online submission form on your behalf.

Please know it is PLOS ONE policy for corresponding authors to declare, on behalf of all authors, all potential competing interests for the purposes of transparency. PLOS defines a competing interest as anything that interferes with, or could reasonably be perceived as interfering with, the full and objective presentation, peer review, editorial decision-making, or publication of research or non-research articles submitted to one of the journals. Competing interests can be financial or non-financial, professional, or personal. Competing interests can arise in relationship to an organization or another person. Please follow this link to our website for more details on competing interests: <http://journals.plos.org/plosone/s/competing-interests>

**Response:** According to the Journal Requirements we included the required sentence in the Declaration of interests’ section.

5. In your Data Availability statement, you have not specified where the minimal data set underlying the results described in your manuscript can be found. PLOS defines a study's minimal data set as the underlying data used to reach the conclusions drawn in the manuscript and any additional data required to replicate the reported study findings in their entirety. All PLOS journals require that the minimal data set be made fully available. For more information about our data policy, please see <http://journals.plos.org/plosone/s/data-availability>.

Upon re-submitting your revised manuscript, please upload your study’s minimal underlying data set as either Supporting Information files or to a stable, public repository and include the relevant URLs, DOIs, or accession numbers within your revised cover letter. For a list of acceptable repositories, please see [http://journals.plos.org/plosone/s/data-availability#loc-recommended-repositories](http://journals.plos.org/plosone/s/data-availability#loc-recommended-repositories). Any potentially identifying patient information must be fully anonymized.

Important: If there are ethical or legal restrictions to sharing your data publicly, please explain these restrictions in detail. Please see our guidelines for more information on what we consider unacceptable restrictions to publicly sharing data: [http://journals.plos.org/plosone/s/data-availability#loc-unacceptable-data-access-restrictions](http://journals.plos.org/plosone/s/data-availability#loc-unacceptable-data-access-restrictions). Note that it is not acceptable for the authors to be the sole named individuals responsible for ensuring data access. We will update your Data Availability statement to reflect the information you provide in your cover letter.

**Response:** According to the Journal Requirements we had fully rewritten the Data availability section.

6. Your ethics statement must appear in the Methods section of your manuscript. If your ethics statement is written in any section besides the Methods, please move it to the Methods section and delete it from any other section. Please also ensure that your ethics statement is included in your manuscript, as the ethics section of your online submission will not be published alongside your manuscript.

**Response:** According to the Journal Requirements we had fully rewritten the Ethical approval and consent to participate section and moved this section in the Methods section of our manuscript.

7. Please include captions for your Supporting Information files at the end of your manuscript, and update any in-text citations to match accordingly. Please see our Supporting Information guidelines for more information: <http://journals.plos.org/plosone/s/supporting-information>.

**Response:** According to the Journal Requirements we had fully rewritten the Legends of Figures section, and included at the end of the manuscript the Supporting Information section.
