# Supplementary material for: Rehospitalization for pneumonia after first pneumonia admission: Incidence and predictors in a population-based cohort study
Source: PLoS One. 2020 Jun 30;15(6):e0235468. doi: 10.1371/journal.pone.0235468 (PMC7326167; doi:10.1371/journal.pone.0235468)
Supplement: Attachment — Submitted filename: Response to reviewers 10.06.doc [file pone.0235468.s003.doc]

**Academic Editor:** In your Discussion, please remove any reference to the late pneumonia readmissions. This is never really addressed earlier in the manuscript (at least not now with the changes) so I think it should now be removed from here as well. This modification will also require some changes to the paragraph starting line 20 on page 14.

We thank the Academic Editor for this suggestion. We changed the text accordingly.

**Reviewer #1:** This paper has improved substantially focusing the analysis on patients readmitted within 30 days.
The authors have adequately addressed the reviewer comments.
Minor critiques:
The authors should review abstract, objective does not correspond with the main text (predictors of late readmissions were not evaluated).

We thank the reviewer for this suggetion. We changed the text accordingly.

**Reviewer #2:** My comments were addressed sufficiently and the paper now appears to be appropriate for publication in my opinion.

Thank you
